# Crosstalk between Peroxisomal Activities and Nrf2 Signaling in Porcine Embryos

**DOI:** 10.3390/antiox10050771

**Published:** 2021-05-13

**Authors:** Eui-Hyun Kim, Muhammad-Rosyid Ridlo, Byeong-Chun Lee, Geon A. Kim

**Affiliations:** 1 Department of Theriogenology and Biotechnology, Research Institute for Veterinary Science, College of Veterinary Medicine, Seoul National University, Seoul 08826, Korea; hyun9214@snu.ac.kr (E.-H.K.); rosyidridlodrh@gmail.com (M.-R.R.); bclee@snu.ac.kr (B.-C.L.); 2 Optipharm Inc., Cheongju 28158, Korea; 3 Department of Bioresources Technology and Veterinary, Vocational College, Universitas Gadjah Mada, Yogyakarta 55281, Indonesia; 4 Department of Clinical Pathology, College of Health Science, Eulji University, Uijeongbu 11759, Korea

**Keywords:** porcine embryos, IVC, melatonin, phytanic acid, Nrf2 signaling, lipid metabolism

## Abstract

Melatonin and phytanic acid (PA) are known to be involved in lipid metabolism and β-oxidation, in which peroxisomal activities also significantly participate. In addition, other studies have reported that the nuclear factor-erythroid-derived 2-like 2 (Nrf2 or NFE2L2) signaling pathway mediates lipid metabolism and its subsequent cascades. As these mechanisms are partially involved in porcine oocytes or embryonic development, we hypothesized that the factors governing these mechanisms could be interconnected. Therefore, we aimed to investigate possible crosstalk between peroxisomal activities and Nrf2 signaling in porcine embryos following melatonin and PA treatment. Porcine embryos were cultured for seven days after parthenogenetic activation, and subsequently treated with melatonin and PA, or injected with *Pex19*-targeted siRNAs. Real-time PCR, immunocytochemistry, and BODIPY staining were used to evaluate peroxisomal activities, Nrf2 signaling, and subsequent lipid metabolism. We found that melatonin/PA treatment enhanced embryonic development, whereas injection with *Pex19*-targeted siRNAs had the opposite effect. Moreover, melatonin/PA treatment upregulated peroxisomal activities, Nrf2 signaling, lipid metabolism, and mitochondrial membrane potentials, whereas most of these mechanisms were downregulated by *Pex19*-targeted siRNAs. Therefore, we suggest that there is a connection between the action of melatonin and PA and the Nrf2 signaling pathway and peroxisomal activities, which positively influences porcine embryonic development.

## 1. Introduction

The production of transgenic pigs and xenotransplantation are pivotal in translational biomedical research [1,2], and the development of optimal procedures for embryo production are essential [3]. However, overcoming the four-cell block and maximizing the developmental potentials of porcine embryos still remains an unsolvable assignment for researchers [4,5]; therefore, attempts to improve the porcine IVC system and transgenic pig production using either chemical treatment or assistive reproductive technologies (ARTs) have been continuously reported [6,7,8,9]. Interestingly, both phytanic acid (PA) (a branched fatty acid (FA) and melatonin (a mammalian pineal gland- and peripheral reproductive organ-derived natural hormone) are suggested to be involved in lipid metabolism in mammalian cells [10,11], oocytes [12,13,14], and embryos [7,15,16]. Although there has been a study of their opposite mechanisms in cells [17], there have been no reports on their synergistic effect on lipid metabolism, specifically in mammalian oocytes or embryos.

It is widely accepted that the Nrf2 signaling pathway is involved in cellular defense mechanisms against oxidative stress. Under cellular oxidative stress conditions, Nrf2 detaches from Keap1 through the breakage of disulfide bonds, stabilizes itself, and translocates into the nucleus to facilitate ARE activation [18,19]. The action of this pathway in mammalian oocytes [20,21,22] and embryos [7,21,23] has previously been proven; moreover, some of these studies suggested that the potential for embryonic development and maturation also increased with pathway activation. In addition, the study by Van Den Brink and Wanders et al. suggested that α-oxidation from the digestion of PA occurs solely in peroxisomes [24]. Due to the branched structure of PA, it undergoes a series of shortening processes via phytanoyl-CoA hydroxylase (PHYH)-mediated α- and β-oxidation [25], and the subsequent short-chained FAs are transferred to the mitochondria to facilitate ATP production via the citric acid cycle [26]. Interestingly, there have been some studies on the role of β-oxidation in mammalian oocytes or embryos that claim that FA is one of the most pivotal sources of energy for the development of oocyte competence and embryonic developmental potentials [27,28,29].

Pex19 is a peroxisome biogenesis factor that collaborates with Pex3. This collaboration is known to participate in the categorization of membrane proteins for transportation to other organelles, such as lipid droplets and mitochondria [30]. Sunyer-Figueres et al. reported that melatonin significantly increased the expression of Pex19, suggesting that melatonin upregulates β-oxidation of FAs in peroxisomes through the regulation of Pex19 [31]. Here, some studies raised an interesting suggestion that the nuclear factor-erythroid-derived 2-like 2 (Nrf2 or NFE2L2) signaling pathway not only plays a role in the maintenance of cellular redox homeostasis, but also mediates the β-oxidation of FAs, mitochondrial membrane potential (MMP), and the production of ATP. Moreover, it has been reported that the Nrf2 signaling pathway has a beneficial effect on compromised mitochondria [32,33].

To date, there have been numerous emerging suggestions on the co-regulation of the Nrf2 signaling pathway and peroxisomal activity via the activities of Pex19 and PHYH; however, we could not find any studies on their relationship in porcine embryos. Since both the Nrf2 signaling pathway and peroxisomal activities are found to be involved in β-oxidation and lipid metabolism in eukaryotic cells, we suggest that their crosstalk in porcine embryos needs to be investigated, as it may prove to be a significant key to improving porcine IVC. Therefore, the aim of this study was to investigate whether the regulation of peroxisomal activities would affect the Nrf2 signaling pathway and subsequent lipid metabolism in porcine embryos treated with melatonin and PA.

## 2. Materials and Methods

### 2.1. Research Ethics and Chemicals

Porcine ovaries were delivered from a local abattoir after manufacturing processes. The Institutional Animal Care and Use Committee (IACUC) of Seoul National University approved the usage of ovaries (Approval ID: SNU-190621-2-1). All chemicals and reagents used in this study were obtained from Sigma-Aldrich, unless otherwise indicated.

### 2.2. In Vitro Maturation

The entire process of porcine IVM has been previously described [34]. In brief, ovaries from a local abattoir were collected and delivered to the laboratory. Cumulus-oocyte complexes (COCs) were recovered by aspiration with 10 mL syringes. The COCs were washed three times in washing medium and selected in accordance with three categories: distinct cellular membrane, homogenous cytoplasm, and more than three layers of cumulus cells (CCs). The selected COCs were transferred to IVM medium and incubated at 39 °C under 5% CO_2_ in 95% humidified air. After 20–22 h of maturation culture with hormones, the COCs were cultured again in IVM medium without hormones for an additional 20–22 h.

### 2.3. Parthenogenetic Activation

The process of parthenogenetic activation has been described previously [7]. Denuded oocytes were activated in activation medium, comprising 0.28 M mannitol, 0.1 mM CaCl_2_, 0.1 mM MgSO_4_, and 0.5 mM HEPES, using 60 μs electrical stimulation with a direct current pulse of 1.5 kV/cm using a BTX Electro-Cell Manipulator 2001 (BTX Inc., San Diego, CA, USA) in a 3.2 mm double electrode chamber. The activated oocytes were subsequently washed and stabilized in porcine zygote Medium-5 (PZM-5; CSR-CK024; Waco Chemicals, Osaka, Japan). Finally, they were cultured in 40 μL droplets of PZM-5, covered with mineral oil, and incubated at 39 °C in a humidified atmosphere of 5% CO_2_, 5% O_2_, and 90% N_2_ for 7 days.

### 2.4. Microinjection

Matured oocytes were denuded with 0.1% hyaluronidase, and then parthenogenetically activated. After 1 h of culture, potential embryos were microinjected into 4 μL droplets of 7.5 μg/mL cytochalasin B using an Eppendorf FemtoJet microinjector. Candidate (Cnd) siRNAs targeting *Pex19* and scramble siRNA [35] for the negative control were designed and synthesized by Bioneer Co. (Daejeon, Korea). Information on these siRNAs is shown in Table 1. For injection, 25 μmol/L siRNA was loaded onto FemtoTip II (Eppendorf) using a microloader (Eppendorf), and injection of the siRNA was confirmed by visualization of the distinct expansion of droplets within the cytoplasm of the embryo. Immediately after microinjection, the embryos were washed several times in PZM-5 droplets and cultured for 7 days.

### 2.5. Chemical Administration during IVC and Embryo Evaluation

After parthenogenetic activation or microinjection, cells were treated with a series of PA concentrations during IVC: 0 (control), 20, 40, and 80 μM, to determine the optimum concentration. For melatonin, we used concentrations of 10^−7^ M melatonin, as this was demonstrated to be the optimal concentration in a previous study. Both PA and melatonin were dissolved in DMSO, and for exact comparison, the control and microinjected groups were also treated with DMSO. The day on which the activated or microinjected oocytes were transferred to the IVC medium was denoted as day 0. The number of embryos with even cleavage was counted under a stereomicroscope on day 2 (at 48 h). On day 7, the number of blastocysts and total cell numbers were counted (168 h). The blastocysts from day 7 were washed in PVA/PBS and then fixed for 1 h in 4% paraformaldehyde (*w*/*v*) in PBS at room temperature. Subsequently, the day 2 embryos and blastocysts were stained with 5 μg/mL Hoechst 33,342 for 8 min. After washing with PVA/PBS, the stained embryos were mounted on glass slides and covered with cover slips. The total cell numbers of the day 2 and day 7 embryos were determined under a fluorescence microscope (Nikon Corp., Tokyo, Japan). In each group, at least 15 embryos from five biological replications were used for staining.

### 2.6. Immunofluorescence Staining

Day 2 and 7 (two-cell and blastocyst, respectively) embryos were washed in PVA/PBS and then fixed with 4% paraformaldehyde (PFA) (*w*/*v*) in PBS for 1 h at room temperature. The embryos were permeabilized with 1% Triton X-100 (*v*/*v*) in distilled water (DW) for 1 h at 39 °C, washed three times in 1% PVA/PBS, and then incubated in 2% BSA in PVA/PBS for 2 h to prevent non-specific binding. The embryos were then directly transferred to 2% BSA containing the primary antibody for NRF2 (1:200; 70R-50116; Fitzgerald Industries International, Acton, USA), PHYH (1:400; MBS3212923; MyBioSource, San Diego, CA, USA), and PEX19 (1:400; MBS9605735; MyBioSource, San Diego, CA, USA) and incubated overnight at 4 °C. After incubation, the embryos were washed several times in PVA/PBS and incubated with secondary fluorescein isothiocyanate-conjugated anti-rabbit polyclonal antibody (1:200; ab6717; Abcam, Cambridge, UK) and goat anti-rabbit IgG H&L (Texas Red ^®^, 1:200; ab6719; Abcam, Cambridge, UK) at 37 °C for 2 h in the dark. After the secondary antibody incubation, the embryos were washed in PVA/PBS and the counterstaining was directly performed with 5 μg/mL Hoechst-33342 for 8 min. After washing, embryos were mounted on glass slides with 100% glycerol droplets, covered with cover slips, and then analyzed under a fluorescence microscope. Assessment of the fluorescence was performed using ImageJ software (version 1.46r; National Institute of Health, MD, USA). The intensities of the control group were standardized to 1. Staining was performed at least three times, and in each group, at least 15 embryos from eight biological replications were used for the staining.

### 2.7. Fluorescent FA Analog Assays

The fluorescent FA analog assay was performed in accordance with a study by Lolicato et al. [36]. In brief, day 2 and day 7 embryos were washed fixed in 4% PFA/PBS for 1 h at room temperature, then washed three times in PVA/PBS. The fixed embryos were then incubated in 6 μM BODIPY 558/568 C12 (BODIPY-FA; D3835; Molecular Probes, Eugene, OR, USA) diluted in PBS for 1 h at room temperature (light avoided). After incubation, the embryos were again washed three times in PVA/PBS before mounting on glass slides with cover slips. Images were captured using an epifluorescence microscope (TE2000-S; Nikon) and the fluorescence intensities of the FA were evaluated using ImageJ software. The intensities of the control group were standardized to 1. The staining was performed at least three times technically and, in each group, at least 15 embryos from eight biological replications were used for the staining.

### 2.8. JC-1 MMP Assays

The entire procedure of the JC-1 MMP assay was previously described [12]. Day 2 and 7 embryos were fixed and washed in PVA/PBS, and then incubated at 37 °C in JC-1 solution mixed with culture medium (PZM-5) for 30 min. After incubation, the embryos were washed with PVA/PBS several times before mounting on cover slips. Images of each embryo were obtained using an epifluorescence microscope (TE2000-S; Nikon). The fluorescence ratio of the JC-1 aggregate and JC-1 monomer (590 nm and 530 nm, respectively) was measured using ImageJ software (version 1.46r; National Institutes of Health, MD, USA). The intensities of the control group were standardized to 1. Staining was technically performed at least three times, and in each group, at least 17 embryos from five biological replications were used for the staining.

### 2.9. ATP Content Assay

Day 2 and 7 embryos were washed in PVA/PBS three times, and then fixed in 4% PFA/PBS for 1 h at room temperature. The fixed embryos were washed three times in PVA/PBS droplets, and then transferred to 0.5 μM of BODIPY FL ATP (BODIPY-ATP; A12410; Molecular Probes, Eugene, OR, USA) diluted in PBS for 1 h at room temperature. Subsequently, the embryos were washed again in PVA/PBS and mounted on glass slides and covered with slips. Images were captured using an epifluorescence microscope, and the ATP content was measured using ImageJ software. The staining was technically performed three times, and in each group, at least 21 embryos from five biological replicates were used for the staining.

### 2.10. Analysis of Gene Expression by Quantitative Real-Time PCR

The procedure for quantitative real-time PCR (qRT-PCR) has been previously described [9]. In brief, total RNA was extracted from frozen day 2 and day 7 embryos and subsequently synthesized to complementary DNA (cDNA). For qRT-PCR, reaction mixtures contained 10 μL SYBR Premix Ex Taq (Takara, Otsu, Japan), 0.4 μL (10 pmol/μL) each of forward and reverse primers, 8.2 μL of nuclease-free water (NFW), and 1 μL cDNA in a PCR plate (Micro-Amp Optical 96-Well Reaction Plate, Applied Biosystems, Singapore). Primer sequences are listed in Table 2. Amplification was performed using the Applied Biosystems StepOneTM Real-Time PCR System (Applied Biosystems, Waltham, MA, USA). A total of 40 reaction cycles were performed, consisting of denaturation for 15 s at 95 °C, annealing for 1 min at 60 °C, and 1 min of extension at 72 °C. The experiment was technically performed four times, and in each group, at least 200 day 2 embryos and 60 day 7 embryos from more than 10 biological replications were used. Target gene expression was quantified relative to that of the endogenous control gene (GAPDH). The relative expressions were calculated using the following equation [37]:

R = 2^−[ΔCt sample − ΔCt control]^

### 2.11. Statistical Analysis

All experiments were performed at least three times. All statistical analysis was performed using GraphPad PRISM 5.01 (PRISM 5, GraphPad Software, Inc., San Diego, CA 92108, USA). Data are expressed as the mean ± SEM in order to determine significant differences among experimental groups and analyzed by univariate analysis of variance (ANOVA) with a Tukey’s post hoc multiple comparison test. *p*-values < 0.05 were considered to be significantly different among the experimental groups.

## 3. Results

### 3.1. Optimization of PA and Co-Treatment with Melatonin

**Figure 1:**
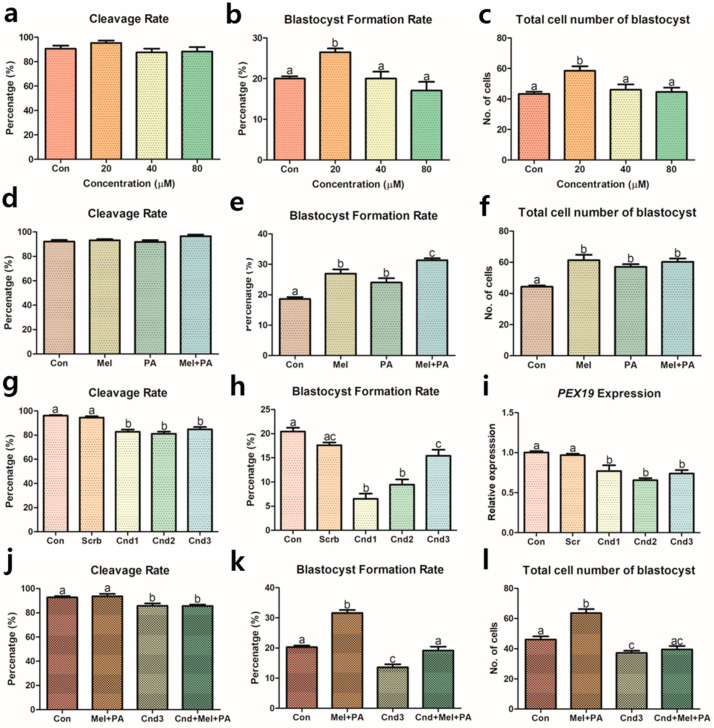
Assessments of porcine embryonic development treated with melatonin and phytanic acid (PA), and injected with *Pex19*-targeted siRNAs. (**a**–**c**) Optimization of PA during porcine IVC through rates of cleavage, blastocyst formation, and total cell numbers of blastocysts. (**d**–**f**) Assessment of co-treatment effect by melatonin and PA. (**g**,**h**) Selection of *Pex19*-targeted siRNAs through embryonic development and (**i**) verification of *Pex19* knockdown by real-time PCR. (**j**–**l**) Assessment of melatonin/PA treatment and siRNA on porcine embryos through rates of cleavage, blastocyst formation, and total cell numbers of blastocysts. At least five biological replications were performed and more than 170 embryos from each experimental group were used. Data are shown as the means ± SEM. Groups marked with different alphabetical letters are significantly different (*p* < 0.05). Con, control; mel, melatonin; PA, phytanic acid; Cnd, candidate.

Different concentrations of PA (control (0), 20, 40, and 80 μM) were tested to determine the appropriate concentrations for porcine IVC. After seven days of embryo culture, the cleavage rate, blastocyst formation rate, and total blastocyst cell number were evaluated. None of the experimental groups showed any differences in cleavage rates (Figure 1a). However, treatment with 20 μM PA resulted in a higher blastocyst formation rate compared to the control, 40, and 80 μM treated groups (26.48% vs. 20.00%, 20.00%, and 17.06%, respectively; *p* < 0.05) (Figure 1b). Moreover, 20 μM PA showed the highest total cell number of blastocysts compared to the control, 40, and 80 μM treated groups (58.50 vs. 43.31%, 46.10%, and 44.57%, respectively; *p* < 0.05) (Figure 1c). Therefore, 20 μM PA was considered the optimal concentration for porcine IVC. A total of five biological replications were performed, and more than 170 porcine embryos per experimental group were used.

Treatment with 20 μM PA was used in subsequent experiments in which the embryos were co-treated with melatonin and PA. There were no differences in the rates of embryo cleavage among the experimental groups (Figure 1d). However, the individually treated melatonin and PA groups had significantly higher blastocyst formation rates than the control group (26.96% and 24.02% vs. 18.63%, respectively; *p* < 0.05) (Figure 1e). Furthermore, the melatonin and PA co-treated group showed the highest blastocyst formation rate compared to all other experimental groups (31.35%, *p* < 0.05). As shown in Figure 1f, the total cell number of blastocysts in the melatonin-, PA-, and co-treated groups were significantly higher than that of the control group (61.30, 57.08, 60.31, and 44.30%, respectively; *p* < 0.05). More than 180 embryos were used per experimental group, with six biological replications each. Presumably, this result might indicate that co-treatment with melatonin and PA was more effective than either individual treatment.

### 3.2. Pex19 siRNA Selection and Application

To select an appropriate *Pex19* targeting siRNA, three Cnds were designed and used for microinjection. In Figure 1g, differences in cleavage rates were observed between the scramble siRNA and all Cnds (Cnd1, 2, and 3); rates in the Cnd-treated groups were significantly lower than those in the control and scramble siRNA groups (82.76%, 81.16%, and 84.82% vs. 96.10% and 94.48%, respectively; *p* < 0.05). All Cnd-treated embryos (Cnd1, 2, and 3) had significantly lower blastocyst formation rates than either the control or scramble siRNA groups (6.52%, 9.45%, and 15.40% vs. 20.42% and 17.59%, and 17.06%, respectively; *p* < 0.05) (Figure 1h). However, there were no differences between the scrambled siRNA and Cnd3. In addition, we analyzed the gene expression of *Pex19* in porcine two-cell embryos, and found that all Cnds significantly reduced *Pex19* expression compared to the control and scramble siRNA (Figure 1i) (*p* < 0.05). From this experiment, Cnd3 was selected as the appropriate siRNA for subsequent experiments because it significantly reduced the expression of *Pex19*, but had the highest blastocyst formation rate, indicating that Cnd3 was the least harmful to embryonic development. At least 180 embryos were used for each experimental group, and six biological replications were performed. For gene (*Pex19*) expression analysis, a total of 844 two-cell embryos from five biological replications were used for total RNA extraction, and real-time PCR was performed in triplicate.

With the selected siRNA, the last test for IVC was performed and the experimental groups were as follows: control (DMSO treated), melatonin/PA, Cnd3, and Cnd3 with melatonin/PA (combination group). In Figure 1j, the cleavage rates in the Cnd3 and combination groups were significantly lower than those of the control and melatonin/PA-treated groups (85.77% and 85.67% vs. 92.74% and 93.52%, respectively; *p* < 0.05). The melatonin/PA-treated group showed the highest blastocyst formation rates compared to the control, Cnd3, and combination groups (31.62% vs. 20.29%, 13.60%, and 19.19%, respectively; *p* < 0.05), and Cnd3 had the lowest blastocyst formation rate among all groups (*p* < 0.05) (Figure 1k). Similarly, the melatonin/PA-treated group also showed the highest blastocyst cell numbers compared to the control, Cnd3, and the combination groups (63.58 vs. 46.08, 37.15, and 39.58, respectively; *p* < 0.05) (Figure 1l). At least 200 embryos from each experimental group from six biological replications were used.

### 3.3. Gene Expression Analysis in Two-Cell Embryos and Blastocysts

**Figure 2:**
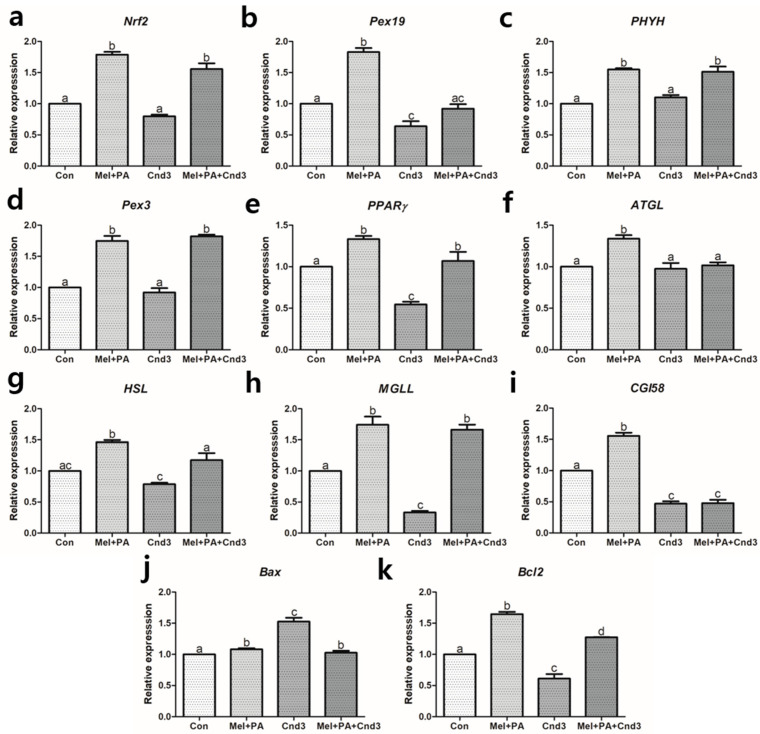
Gene expression evaluations of melatonin/PA treatment and Cnd3-injection on porcine two-cell embryos. The gene expressions of (**a**) Nrf2, the main factor in the Nrf2 signaling pathway, (**b**–**e**) factors that are related to peroxisomal activities, (**f**–**i**) lipid metabolism, and (**j**,**k**) apoptosis in the embryos were analyzed within the four groups. More than 200 embryos in each experimental group from six biological replications were used and real-time PCR was performed three times technically. Within the same mRNA, bars with different alphabetical letters are significantly different among the groups (*p* < 0.05). Con, control; mel, melatonin, PA, phytanic acid; Cnd3, siRNA candidate 3.

**Figure 3:**
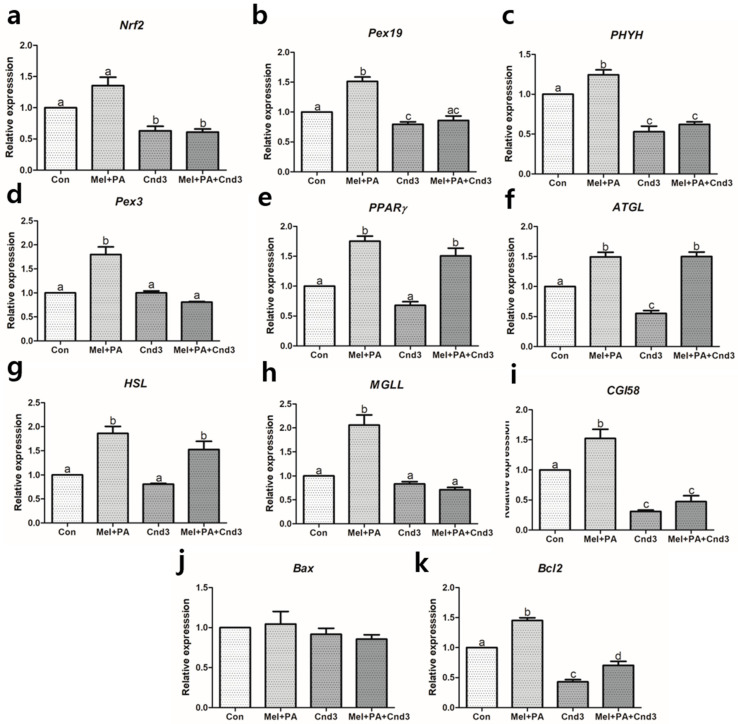
Gene expression evaluations of melatonin/PA treatment and Cnd3-injection on porcine blastocysts. The gene expressions of (**a**) Nrf2, the main factor in the Nrf2 signaling pathway, (**b**–**e**) factors that are related to peroxisomal activities, (**f**–**i**) lipid metabolism, and (**j**,**k**) apoptosis in the embryos were analyzed within the four groups. More than 50 blastocysts in each experimental group from six biological replications were used and real-time PCR was performed three times technically. Within the same mRNA, bars with different alphabetical letters are significantly different among the groups (*p* < 0.05). Con, control; mel, melatonin, PA, phytanic acid; Cnd3, siRNA candidate 3.

Figure 2 and Figure 3 show the gene expression of components of the Nrf2 signaling, peroxisomal activities, lipid metabolism, and apoptosis pathways in porcine two-cell embryos and blastocysts. In two-cell embryos, the expression of *Nrf2* was highly increased when melatonin/PA was applied, regardless of Cnd3 injection (*p* < 0.05), and there was no significant difference in the Cnd3-injected group (Figure 2a). In contrast, in blastocysts, no difference was observed between the control and melatonin/PA-treated groups; however, gene expression in the Cnd3-injected and combination groups showed a significant decrease compared to the other groups (*p* < 0.05; Figure 3a). In the case of *Pex19* (Figure 2b and Figure 3b), melatonin/PA-treatment significantly upregulated gene expression and the Cnd3-injected group showed decreased expression in two-cell embryos (*p* < 0.05). In blastocysts, a similar tendency in expression was observed in the melatonin/PA-treated group (*p* < 0.05), and downregulation was observed in the Cnd3-injected group (*p* < 0.05). In *PHYH* (Figure 2c and Figure 3c), the melatonin/PA-treated group showed significantly increased expression in both types of embryos; however, expression was decreased in the Cnd3 and combination groups compared to the control group in blastocysts (*p* < 0.05). In addition, an increase in the expression was observed in the combination group compared to the control in two-cell embryos (*p* < 0.05).

In two-cell embryos, melatonin/PA treatment significantly upregulated the expression of *Pex3*, *PPARγ*, *ATGL*, *HSL*, *MGLL*, *CGI58*, and *Bcl2*, whereas the expression of *PPARγ*, *HSL*, *MGLL*, and *Bcl2* was the lowest in the Cnd3-injected group (*p* < 0.05). Interestingly, expression of *Pex3*, *PPARγ*, *HSL*, *MGLL*, and *Bcl2* was recovered in the combination group compared to the Cnd3-injected group (*p* < 0.05). The gene expression of *Bax* was opposite that of *Bcl2* in Cnd3 and the combination group (*p* < 0.05) (Figure 2d–k). The gene expression signatures in blastocysts (Figure 3d–k) were similar to those observed in the two-cell embryos; melatonin/PA treatment significantly increased the expression of *Pex3*, *PPARγ*, *ATGL*, *HSL*, *MGLL*, *CGI58*, and *Bcl2*, whereas only *ATGL*, *CGI58*, and *Bcl2* showed a significant decrease in gene expression in the Cnd3-injected group (*p* < 0.05). Moreover, recovery of the gene expression of *PPARγ*, *ATGL*, *HSL*, and *Bcl2* was observed in the combination group compared to the Cnd3-injected group (*p* < 0.05).

### 3.4. Immunocytochemistry, BODIPY, and JC-1 MMP Staining in Porcine Embryos

**Figure 4:**
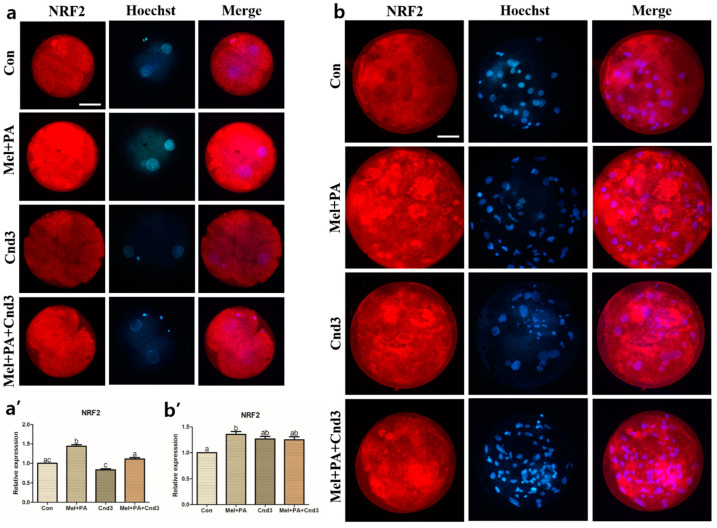
Representative images of immunocytochemical analysis of NRF2 in porcine two-cell embryo blastocysts. Fluorescence microscopy was applied to obtain images of embryos. (**a**,**b**) Representative two-cell embryos in each group were stained with NRF2 rabbit-derived antibody and counterstained with Hoechst 33342. (**a’**,**b’**) Analysis of intensities from NRF2-stained two-cell embryos and blastocysts. At least 20 two-cell embryos and 15 blastocysts from five biological replications in each group were used for the staining and immunocytochemistry was performed three times technically. Data are shown as the means ± SEM. Bars with different alphabetical letters are significantly different (*p* < 0.05). Con, control; mel, melatonin, PA, phytanic acid; Cnd3, siRNA candidate 3. White bars in the images indicate 50 µm; 400× magnification.

**Figure 5:**
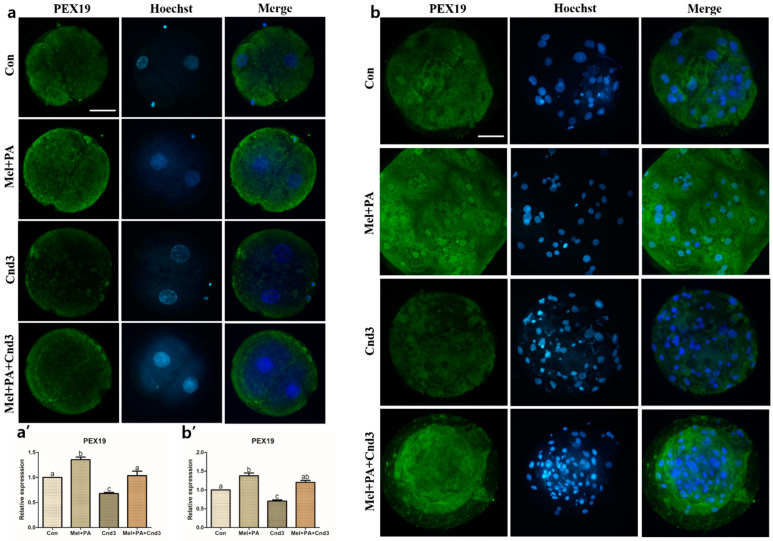
Representative images of immunocytochemical analysis of PEX19 in porcine two-cell embryo blastocysts. Fluorescence microscopy was applied to obtain images of embryos. (**a**,**b**) Representative two-cell embryos in each group were stained with PEX19 rabbit-derived antibody and counterstained with Hoechst 33342. (**a’**,**b’**) Analysis of intensities from PEX19-stained two-cell embryos and blastocysts. At least 20 two-cell embryos and 15 blastocysts from five biological replications in each group were used for the staining and immunocytochemistry was performed three times technically. Data are shown as the means ± SEM. Bars with different alphabetical letters are significantly different (*p* < 0.05). Con, control; mel, melatonin, PA, phytanic acid; Cnd3, siRNA candidate 3. White bars in the images indicate 50 µm; 400× magnification.

**Figure 6:**
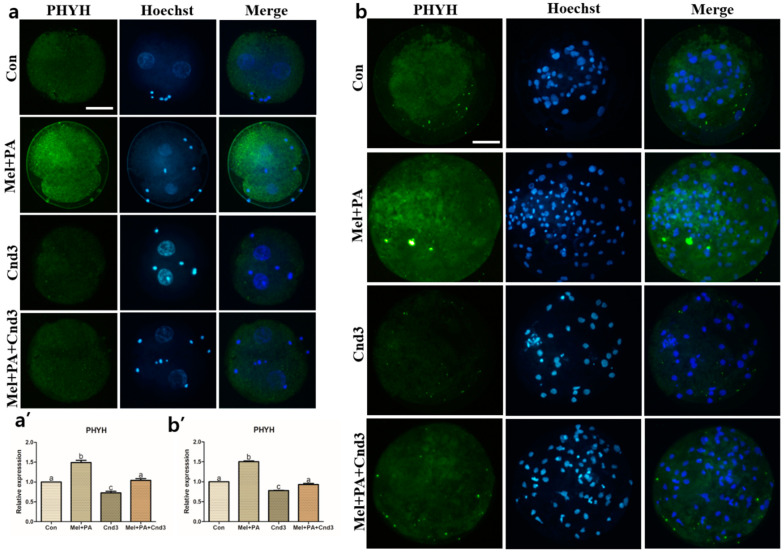
Representative images of immunocytochemical analysis of PHYH in porcine two-cell embryo blastocysts. Fluorescence microscopy was applied to obtain images of embryos. (**a**,**b**) Representative two-cell embryos in each group were stained with PHYH rabbit-derived antibody and counterstained with Hoechst 33342. (**a’**,**b’**) Analysis of intensities from PHYH-stained two-cell embryos and blastocysts. At least 20 two-cell embryos and 15 blastocysts from five biological replications in each group were used for the staining and immunocytochemistry was performed three times technically. Data are shown as the means ± SEM. Bars with different alphabetical letters are significantly different (*p* < 0.05). Con, control; mel, melatonin, PA, phytanic acid; Cnd3, siRNA candidate 3. White bars in the images indicate 50 µm; 400× magnification.

Immunocytochemistry of the protein of interest was performed on porcine two-cells and blastocysts. Figure 4, Figure 5 and Figure 6 show the protein expression of NRF2, PEX19, and PHYH in two-cell embryos; expression was significantly increased in the melatonin/PA-treated group, whereas a decrease was observed in the Cnd3-injected group compared to the control group (*p* < 0.05). The expression was again significantly improved in the combination group compared to the Cnd3-injected group (a and a’ in the figures indicate results from two-cell embryos). Similarly, in blastocysts, melatonin/PA treatment significantly upregulated protein expression. However, only PEX19 and PHYH protein expression were significantly decreased in the Cnd3-injected group compared to the control group, and recovery of protein expression was observed in the combination group compared to the Cnd3 alone-injected group (*p* < 0.05). In addition, the expression of NRF2 in the combination group was significantly higher than that in the control group (*p* < 0.05), and no difference was observed between Cnd3 and the combination groups (b and b’ in the figures indicate the results from blastocysts).

**Figure 7:**
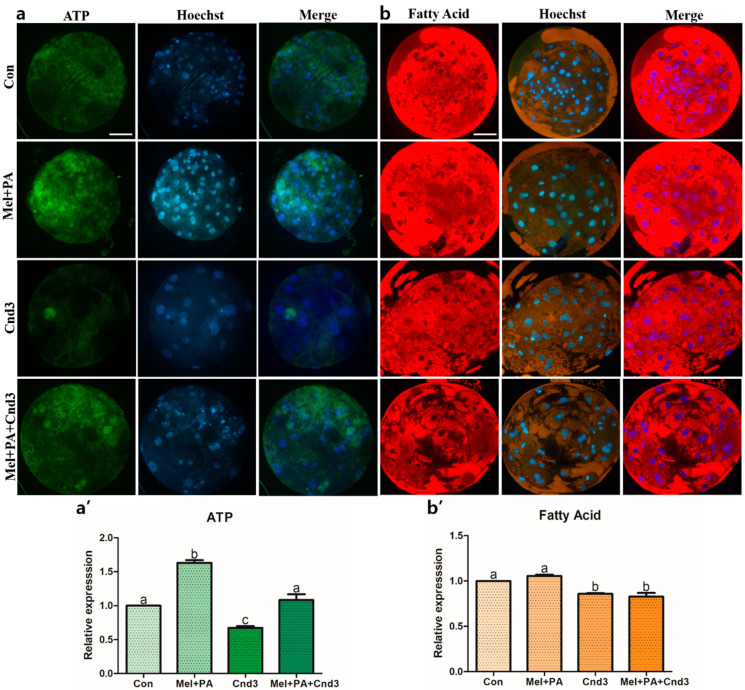
Representative images of BODIPY staining analysis of ATP contents and fatty acids in blastocysts. Fluorescence microscopy was applied to obtain images of the blastocysts. (**a**,**b**) Representative blastocysts in each group were stained with BODIPY FL ATP and BODIPY 558/568 C^12^, respectively, then counter-stained with Hoechst 33342. (**a’**,**b’**) Analysis of intensities from BODIPY FL ATP- and BODIPY 558/568 C^12^-stained blastocysts. At least 15 blastocysts from four biological replications in each group were used for the staining and it was performed three times technically. Data are shown as the means ± SEM. Bars with different alphabetical letters are significantly different (*p* < 0.05). Con, control; mel, melatonin, PA, phytanic acid; Cnd3, siRNA candidate 3. White bars in the images indicate 50 µm; 400× magnification.

**Figure 8:**
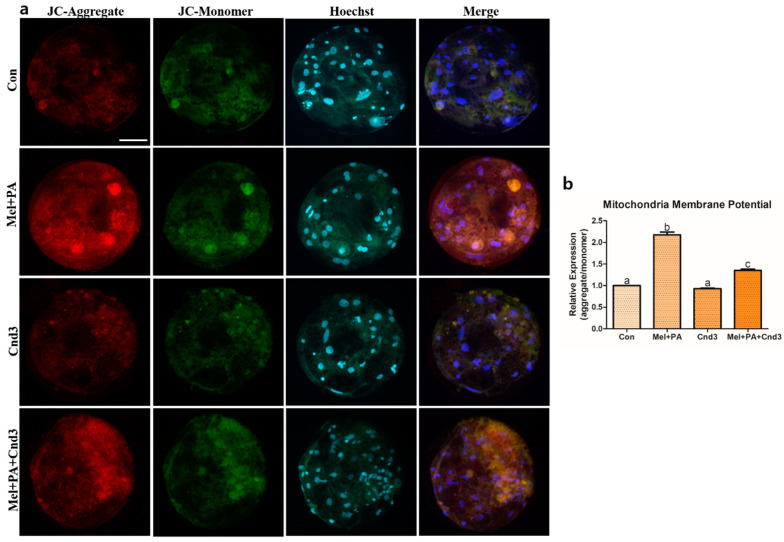
Representative images of JC-1 MMP staining in blastocysts. Fluorescence microscopy was applied to obtain images of the blastocysts. (**a**) Representative blastocysts in each group were stained with JC-1 aggregate and JC-1 monomer, then counter-stained with Hoechst 33342. (**b**) Analysis of intensities from JC-1 MMP-stained blastocysts. At least 15 blastocysts from four biological replications in each group were used for the staining and it was performed three times technically. Data are shown as the means ± SEM. Bars with different alphabetical letters are significantly different (*p* < 0.05). Con, control; mel, melatonin, PA, phytanic acid; Cnd3, siRNA candidate 3. White bars in the images indicate 50 µm; 400× magnification.

Subsequently, ATP, FA, and JC-1 MMP were analyzed in porcine two-cell embryos and blastocysts. The intensity of ATP content was higher in the melatonin/PA-treated group than in the other experimental groups (*p* < 0.05), and Cnd3-injection significantly decreased the intensity in the embryos compared to the control. Lastly, intensity was again increased in the combination group compared to the Cnd3-injected group (*p* < 0.05; Figure 7a,a’). We next investigated the fluorescence of FAs in blastocysts, and found no significant difference in the intensities between the control and melatonin/PA-treated group; however, differences were observed in the Cnd3-injected and combination groups compared to the control and melatonin/PA-treated group (Figure 7b,b’). In addition, JC-1 MMP analysis was performed in blastocysts, and the intensities were calculated from the ratios of JC-1 aggregates/JC-1 monomer. Although there was no difference observed between the control and Cnd3-injected groups, the intensities of the melatonin/PA-treated group and the combination group were significantly higher than those of the control group; melatonin/PA treatment resulted in the highest intensity among the groups (*p* < 0.05; Figure 8a,b).

## 4. Discussion

Many studies have shown the significance of different signaling pathways in mammalian embryonic development. It is generally accepted that the culture conditions and environmental factors must be well controlled when culturing in vitro embryos [38], and it has also been reported that organelle function and epigenetic reprogramming are pivotal in mammalian embryonic development [9,39]. Specific investigations of signaling pathways involved in embryonic development are therefore important in understanding this process. We believe that this is the first study to investigate a possible crosstalk between the Nrf2 signaling pathway and peroxisomal activities in porcine embryos, which were artificially regulated by co-treatment with melatonin and PA and *Pex19*-targeted siRNAs. Our results suggest that the regulation of peroxisomal activities with melatonin, PA, and our Cnd3 influenced porcine embryonic development, possibly via the Nrf2 signaling pathway. Moreover, the treatments and siRNA injections also regulated the Nrf2 signaling pathway, peroxisomal activities, lipid metabolism, and ATP production. Thus, these results suggest that the communication between the Nrf2 signaling pathway and peroxisomal activities might be significant to porcine embryo development and subsequent metabolism.

Melatonin is frequently suggested to play a fundamental role in enhancing mammalian oocyte maturation, embryo development, and antioxidant mechanisms, including lipid metabolism and the Nrf2 signaling pathway [7,14,23,40], whereas PA has been suggested to influence β-oxidation in peroxisomes, lipid metabolism, and ATP production [24,41]. Additionally, the effect of PA on β-oxidation in mammalian gametes and embryos has also been reported [12,42,43]. Therefore, porcine embryos were co-treated with melatonin and PA during IVC. We found that co-treatment with melatonin and PA resulted in a higher blastocyst formation rate compared to individual treatment and the control groups (Figure 1e) and that there was significant upregulation in the expression of genes related to lipid metabolism, including MMP, FA, and ATP content. This result may be supported by the study by Dunning et al., which found that shortened FAs derive from long or branched FAs such as PA initiate lipid metabolism and cause energy metabolism [27]. Moreover, our results may also be supported by some studies demonstrating that lipid-derived ATP synthesis from the mitochondria promotes porcine embryonic development [44,45]. Therefore, we could speculate that melatonin and PA exert a synergistic effect during embryonic development, potentiating the embryos to more lipid metabolism-derived energy production.

We found that melatonin/PA treatment improved embryonic development and increased expression of several genes related to the Nrf2 signaling pathway, peroxisome activities, lipid metabolism, and apoptosis. As is widely known, the Nrf2 signaling pathway is involved in the defense mechanism against oxidative stress; however, some studies suggest additional roles of the pathway, such as embryonic cell cycle progression [21] and anti-aging in embryos [22]. Lastly, Amin et al. suggested the possibility of an involvement in lipid metabolism in embryos [23]. Gene expression of Nrf2 and lipid metabolism were found to be upregulated in two-cell embryos and blastocysts; this is supported by the claims by Ludtmann et al. that Nrf2 has a relationship with the β-oxidation of FAs [46], and it has been demonstrated that ATP production correlates with FA oxidation [47], which may possibly be connected with the actions of Nrf2. We have proven that the Nrf2 signaling pathway and lipid metabolism are interconnected in porcine embryos.

In our study, the peroxisome activities and the Nrf2 signaling pathway were upregulated by the combination treatment of melatonin and PA. In order to prove that the combination group affected the activities of Nrf2 and the peroxisome, *Pex19*-targeted siRNA microinjection was applied in this study. Pex19 and Pex3 function in tandem to modulate peroxisome membrane biogenesis, lipid droplet transportation [48], and the functioning of matured peroxisomes [49,50,51]. In addition, PPARs are involved in lipid detection, peroxisomal gene activators [52], and β-oxidation [53]. PPARγ is also involved in peroxisomal biogenesis [54] and mediates embryo implantation and survival [55]. Sunyer-Figueres et al. stated that *Pex19* is regulated by melatonin, implying that melatonin can mediate FA β-oxidation in peroxisomes by *Pex19* regulation [31]. This was corroborated by our finding that the content of FAs in the Cnd3-injected group was significantly lower than that in the control and melatonin/PA-treated groups (Figure 7b’). Moreover, Pex19 and PPARγ are strongly suggested to have a close relationship with Nrf2 signaling [56,57]. Similar gene and protein expression patterns were primarily observed in peroxisomal activities and lipid metabolism in two-cell embryos and blastocysts. This result is corroborated by our hypothesis that *Pex19*-targeted siRNA would decrease such actions and metabolism. Interestingly, the levels of ATP and Nrf2 gene and protein expression were also partially decreased, which may also be supported by previous reports.

According to some of our results, expression of some genes in the Cnd3-injected group were increased or recovered in the blastocyst stage compared to the two-cell embryo stage. We surmised that these results could be due to several reasons; firstly, several studies have shown siRNAs to have a maximum effect duration of seven days [58,59,60]; however, duration may differ depending on the cell type. Moreover, it has been reported that 24–96 h is the ideal period to evaluate the effects of knockdown and functioning of siRNAs in general [61]. For example, Dadi et al. observed that the knockdown effects of growth factor-related siRNAs were significant in early-stage mouse embryos, and that these effects were reduced during the late blastocyst stage [62], which is corroborated by some of our results. It would be affordable if the *Pex19*-targeted siRNA injected into the porcine embryos could last seven days without diminishing its effects, and our study partially demonstrates the influence of peroxisome knockdown ability. However, we suggest that permanent knockdown techniques, such as the use of shRNAs, should also be applied for further studies.

## 5. Conclusions

In this study, we hypothesized that co-treatment with melatonin and PA not only would upregulate the developmental potentials of porcine embryos but also increase the activities of the Nrf2 signaling pathway and peroxisomes. In addition, combination treatment upregulated lipid metabolism, ATP production, and MMP. In order to verify these connections, we applied *Pex19*-targeted siRNA injection into porcine embryos to determine whether downregulation of *Pex19* affects the Nrf2 signaling pathway and subsequent lipid metabolism and ATP production, and found drastic changes in these pathways. Altogether, we suggest a plausible crosstalk between the activities of the Nrf2 signaling pathway and peroxisome in the process of lipid metabolism and ATP production during the development of porcine embryos. To our knowledge, this is the first report on this crosstalk in porcine embryos. Further investigations and assessments should be conducted in other species and cell types in order to elucidate the intricacies of peroxisomal activity and its influence on Nrf2 signaling and lipid metabolism.

## Figures and Tables

**Table 1:** List of candidate siRNAs targeting *Pex19* and scramble siRNA.

Candidate (Cnds)	Target Gene	Sequences (5′-3′) RNA: (A, C, G, U]), DNA: (a, c, g, t)	GC Content	Tm	NGIC Score
-	Scramble	CGAACAGAUAAAGCCGCUGUAAGUA UACUUACAGCGGCUUUAUCUGUUCG	-	-	-
Cnd1	*PEX19*	GAGAUCUCCAGGAGACACU=tt AGUGUCUCCUGGAGAUCUC=tt	0.53	59.96	98
Cnd2	*PEX19*	CGUGACUUUCCCUCAGGUU=tt AACCUGAGGGAAAGUCACG=tt	0.53	59.51	98
Cnd3	*PEX19*	CACUACACCCUCUUACCUU=tt AAGGUAAGAGGGUGUAGUG=tt	0.47	58.66	91.9

**Table 2:** Sequence-specific primers used for quantification of differential expressed transcripts.

Genes	Primer Sequences (5′-3′)	Product Size (bp)	Accession No.
*GAPDH*	F: GTCGGTTGTGGATCTGACCT R: TTGACGAAGTGGTCGTTGAG	207	NM_001206359
*NRF2*	F: GCCCAGTCTTCATTGCTCCT R: AGCTCCTCCCAAACTTGCTC	115	XM_013984303
*PEX3*	F: AATGCATCTTCCTGGGGACG R: ATACTGTCGTCGTGCTTGGG	125	NM_001244185.1
*PEX19*	F: CTCAATCTATCGGGCCCACC R: TAGACGACACTCCTGCCTCA	144	XM_001928869.5
*PPARγ*	F: CCATTCCCGAGAGCTGATCC R: TTTATCCCCACAGACACGGC	192	XM_005669783.3
*ATGL*	F: GACGGTGGCATCTCAGACAA R: TGGATGTTGGTGGAGCTGTC	113	NM_001098605.1
*HSL*	F: GCCTTTCCTGCAGACCATCT R: CACTGGTGAAGAGGGAGCTG	104	NM_214315.3
*MGLL*	F: ACCCCACAGAGTGTCCCATA R: GGGTGTAGCTGAGGGTTTCC	96	XM_013982013.2
*CGI58*	F: TCTTGCTGGGACACAACCTG R: CCAAAGGGTCCTGCAATCCT	220	NM_001012407.1
*BAX*	F: CATGAAGACAGGGGCCCTTT R: CATCCTCTGCAGCTCCATGT	181	XM_003127290
*BCL2*	F: AATGTCTCAGAGCAACCGGG R: GGGGCCTCAGTTCTGTTCTC	193	NM_214285

F, forward primer; R, reverse primer.

## Data Availability

The data is contained within the article.
